# Editorial Department

**Published:** 1862-01

**Authors:** 


Translated from the German.

CLARKSBURG, VA., Oct. 2, 1861.

MR. EDITOR:—Believing that yon will give an insertion to these few lines in your valuable paper, at the urgent solicitation of many of my fellow sufferers and colleagues, I take the liberty to furnish our fellow citizens of Cincinnati, a correct statement of the privations and treatment of our sick soldiers. There are now from two to three hundred sick soldiers in the hospital. Most of them suffer from rheumatism, dangerous fevers, or like diseases engendered by the irregular habits and life to which they have not been accustomed. All these are now under the treatment of the young, energetic and thoroughly qualified Dr. C. K. Winnie, of the U. S. A., who takes all possible pains to alleviate the sufferings of the defenders of our country, and I am happy to add his labors are rewarded by the weekly discharge of from twenty to thirty men, who, though weak from exhaustion, return cured and sound, though nearly starved, to their respective Regiments.

But, alas! this is the first man we have found doing his duty towards the sick soldier from pure motives, unlike others who join the army for the sake of making money only, and who leave the poor sufferers to their own fate. In the hospitals at Sutton it was horrible to see these unfortunates lying scattered upon the floor without receiving proper treatment and medicines, and without hardly ever being asked or examined where and from what they suffered—did not deem it necessary to examine the patients, but contented with asking the Steward, "How are they all this morning?" to which he received the usual reply, "All right," except some had grown seriously worse and some died during the night. If one would drag himself down stairs and see the Doctor, and beg for medicine, he received some powders or pills, which were uniformly administered for every disease, or the consolatory reply, "I have not the proper medicine for your case." These are the patriots who accompany many of our regiments not to take care of, or administer medical aid to the sick soldier in defending the Union, freedom and right, but to enrich themselves by what they wrongfully withhold from them. For instance, they are entitled to the funds realized from hides and tallow sold from the slaughter house, to procure milk, butter, eggs, &c., &c. for the use of the sick, and also to draw money for the rations not dealt out to the sick soldier. The Steward is impressed to practice the closest economy, and the Physician prescribed half rations, bnt we received no benefits, and in case of complaint being made, the complainant was sure to be locked up. The butcher told our Sergeant of the 5th Company, 9th Regiment, he had paid *fifty dollars* for hides and tallow, which was pocketed. In like manner our Steward sold the coffee, sugar, lard, rice, &c. belonging to the 1st German Regiment, to a shoe-maker, of Sutton, and left us, who were ordered to Clarksburg, the day previous to our march, without provisions. The journey to Clarksburg looked like a funeral train, nothing but bacon and bread for the suffering sick; the money for eggs and butter found its way into wrong pockets. On our arrival we were quartered from forty to fifty in a house, in which already lay about one hundred sick, and we had to content ourselves by lying scattered about on the hall floor and similar places.—The next morning our kind physician appeared, and went to work with a will to bring order out of chaos; create comfort and administer proper medicines to the suffering sick, and in a short time many in Sutton who were considered on the verge of the grave improved rapidly, and became convalescent. But even now there are those in government service as stewards, nurses, &c. paid to take care of the sick, who play their thievish tricks behind the Doctor's back, and take care of themselves not only for the present but for the future.

The Doctor allows every sick soldier full rations, and what is not thus drawn, is paid in cash, for which he procures more nourishing food. Government provides suitable nourishment for the sick soldier, but the leeches around the hospital consume it instead of those for whom it is intended.—If complaints are made to the Doctor they know too well how to clear themselves with falsehoods, and the poor patients get no relief. The office, however small, is made to yield as much as possible at the expense of the soldier. It is high time that government should look into this matter, and put a stop to these nefarious practices, and insure fair treatment. After our painful experience in the camp and hospital, we are almost brought to the belief that men are scarce in the United States, who, if appointed to offices of honor and trust, will discharge their duties honorably and faithfully; but honor to whom honor is due; honor to Dr. Charles K. Winne.

With respects in the name of my comrades and myself,

I am very respectfully yours, a member of

9th Reg't, Ohio Volunteers.

NOTE.—We publish the above letter, which appeared in a German paper of Cincinnati, at the request of a Medical friend, who is acquainted with the gross neglect, incompetency and dishonesty often practiced in the Military Camp and Hospital. We do this with pleasure, since so favorable mention is made of our fellow townsman, Dr C. K. Winne. ED JOUR.

